# *Fusarium musae* from Diseased Bananas and Human Patients: Susceptibility to Fungicides Used in Clinical and Agricultural Settings

**DOI:** 10.3390/jof7090784

**Published:** 2021-09-21

**Authors:** Valeria Tava, Anna Prigitano, Paolo Cortesi, Maria Carmela Esposto, Matias Pasquali

**Affiliations:** 1 Dipartimento di Scienze per gli Alimenti, la Nutrizione e l’Ambiente, Università degli Studi di Milano, 20133 Milano, Italy; valeria.tava@unimi.it (V.T.); paolo.cortesi@unimi.it (P.C.); 2 Dipartimento di Scienze Biomediche per la Salute, Università degli Studi di Milano, 20133 Milano, Italy; Anna.Prigitano@unimi.it (A.P.); maria.esposto@unimi.it (M.C.E.)

**Keywords:** azoles, fusariosis, MIC, antifungal treatments, CLSI protocol

## Abstract

*Fusarium musae* belongs to the *Fusarium fujikuroi* species complex. It causes crown rot disease in banana but also keratitis and skin infections as well as systemic infections in immunocompromised patients. Antifungal treatments in clinical and agricultural settings rely mostly on molecules belonging to the azole class. Given the potential risk of pathogen spread from food to clinical settings, the goal of the work was to define the level of susceptibility to different azoles of a worldwide population of *F. musae*. Eight fungicides used in agriculture and five antifungals used in clinical settings (4 azoles and amphotericin B) were tested using the CLSI (Clinical and Laboratory Standards Institute) protocol methodology on 19 *F. musae* strains collected from both infected patients and bananas. The level of susceptibility to the different active molecules was not dependent on the source of isolation with the exception of fenbuconazole and difenoconazole which had a higher efficiency on banana-isolated strains. Minimal inhibitory concentrations (MICs) of the different molecules ranged from 0.12–0.25 mg/L for prochloraz to more than 16 mg/L for tetraconazole and fenbuconazole. Compared to the *F. verticillioides*, *F. musae* MICs were higher suggesting the importance of monitoring the potential future spread of this species also in clinical settings.

## 1. Introduction

Fusariosis is one of the most common mould infections in humans ranging from superficial diseases, such as onychomycosis and keratitis, to disseminated infections, particularly in haematological cancer and neutropenic patients [1]. The genus *Fusarium* comprises at least 200 species that are not only human pathogens, but they have been isolated also from animals, plants and in specific cases also from the surrounding environment [1,2]. Numerous *Fusarium* species have been classified as cross-kingdom pathogens given their ability to jump from one host to a taxonomically distant one. Among those, *Fusarium musae* [3], sister species of *Fusarium verticillioides* in the *Fusarium fujikuroi* species complex [4], is one of the causative agents of crown rot of banana, a devastating postharvest disease [5,6,7]. The late appearance after distribution in consuming countries make signs of disease impossible to notice during harvest and determine significant losses in banana fruits. Recent studies showed that *F. musae* has also been isolated from human patients where it causes nail and eyes lesions as well as systemic infection in immunocompromised patients [8,9]. It is not clear yet how human beings are infected by *F. musae*. Banana fruits probably act as carriers of *F. musae* spores that reach the consumers after shipping in banana-consuming countries where humans acquire the infection [6]. However, it cannot be excluded that humans acquire the infection after traveling to a banana producing country or through unknown plants or other environmental substrates [9]. It is estimated that 10–30% of the total amount of human Fusariosis are caused by *F. verticillioides* of which 7–20% are actually *F. musae* [9,10,11].

*F. musae* has been reported so far as a cause of banana disease in Dominican Republic [5], Hungary [12], “Neotropical” countries [13,14] and Philippines [3], and cause of infections in humans in Italy, Greece [8], France, Belgium [10] and the USA [15] although it is probable that other *Fusarium* infections have gone undetected due to the difficulty of identifying species. Indeed, species classification relies on multigene sequencing. Differences between *F. verticillioides* and *F. musae* include the excision of the fumonisin gene cluster [3].

The lack of consensus regarding treatment protocols for human fusariosis make the infections difficult to treat [16]. Reference methods for in vitro antifungal susceptibility testing are those of Clinical and Laboratory Standards Institute (CLSI) and European Committee on Antimicrobial Susceptibility (EUCAST) [17], but breakpoints (BPs) have not yet been established [18]. So far most of the clinical and agricultural treatments of *Fusarium* infections rely on the use of azoles, imidazoles and triazoles, which act as inhibitors of ergosterol biosynthesis by blocking 14a demethylation with a selectivity for fungal CYP51 [19]. It has been shown that various azole derivatives can show differences in the spectrum of activity and power of action [20], therefore it is important to know the antifungal effectiveness of different antifungal drugs [21]. In particular knowledge on *F. musae* sensitivity to azoles is limited to few strains from clinical samples [10,11,12,13,14,15,16,17,18,19,20,21,22] and no comparative analysis of strains obtained from agricultural and clinical settings is available.

To have a better understanding on the potential treatments useful against *F. musae*, the aim of this work was to assess the level of susceptibility to 13 antifungal drugs (five from clinical settings and 8 from agricultural settings) of 19 *F. musae* strains isolated from patients and infected bananas. Moreover, we aimed to evaluate whether different sources of the strains could determine differences in antifungal susceptibility. Our work identifies the most effective antifungal compounds for *F. musae* population and suggests that *F. musae* has a lower sensitivity to azoles compared to *F. verticillioides*.

## 2. Materials and Methods

### 2.1. Strains Collection

A worldwide collection of *F. musae* strains was analysed together with a set of four strains obtained at the University of Milan. Five strains of *F. musae* (NRRL 25059, NRRL 25673, NRRL 28893, NRRL 28895, NRRL 28897) isolated from banana fruits, and four strains of *F. musae* isolated from human patient (NRRL 43601, NRRL 43604, NRRL 43658, NRRL 43682) were obtained from ARS Culture Collection Database (USA), two strains of *F. musae* isolated from human patient, and one strain isolated from banana fruits (respectively IHEM 19881, IHEM 20180 and MUCL 52574) from the Belgian co-ordinated collections of Microorganisms, and four strains of *F. musae* (ITEM 1121, ITEM 1142, ITEM 1149, ITEM 1250) isolated from banana fruits obtained from the Institute of Science of Food Production, Bari, Italy (Table 1).

### 2.2. DNA Isolation, PCR and Sequencing

*Fusarium* isolates were grown on V8 media (200 mL V8 juice Campbell, Camden, NJ, USA); 2 g/L CaCO₃ Carlo Erba Reagents S.r.l, Cornaredo, Milano, Italy; 15 g/L agar NeoFroxx GmbH, Einhausen, Germany) where a cellophane membrane was previously placed. After 5 days plates were gently scraped with an inoculation loop and mycelia were collected in plastic tubes, stored at −80 °C and lyophilised the day after.

DNA was extracted from lyophilised mycelia following the protocol (200 microL) of the DNeasy Mericon food Kit (Qiagen, Germantown, MD, USA) [24]. Lyophilised mycelia (200 mg) were weighted and placed in a 2 mL microcentrifuge tube in presence of 1 mL food lysis buffer (provided by the kit) and 2.5 μL Proteinase K solution (provided by the kit). Samples were briefly vortexed to ensure complete distribution of the material and incubated in a water bath at 60 °C for 30 min while manually vortex-shaking the samples every two minutes. After incubation, samples were cooled at room temperature on ice to enhance inhibitor precipitation. Centrifugation of microcentrifuge tubes was performed for 5 min at 2500× *g*. The maximum volume of clear supernatant was drawn from each lysis tube without disturbing the precipitate at the bottom of the tube and the supernatant aliquots were combined in one microcentrifuge tube and mixed by pipetting up and down several times to ensure a homogenous solution. A volume of 700 μL of the clear supernatant pool was transferred to a new microcentrifuge tube already containing 500 μL of chloroform. Tubes were vortexed and centrifuged again for 15 min at 14,000× *g*. New microcentrifuge tubes were prepared containing 350 μL of Buffer PB (provided by the kit) and an aliquot of 350 μL of the upper aqueous phase from our samples was added. One QIAquick spin column (provided by the kit) was prepared per each strain by placing them in a 2 mL collection tube. Samples were vortexed for few seconds and then pipetted into the columns. Tubes and columns were centrifuged at 17,900× *g* for 1 min and the flow-through was discarded. An aliquot of 500 μL Buffer AW2 (provided by the kit) was added, and the centrifugation step was repeated. A second centrifuge step was performed with empty QIAquick spin column to dry the membrane. The columns were then transferred to new microcentrifuge tubes and 150 μL of Buffer EB (provided by the kit) was pipetted directly onto the QIAquick membrane, samples were incubated at room temperature for 1 min and centrifuged at 17,900× *g* for 1 min to elute the final extracted DNA. Samples were separated by electrophoresis onto a 1% (*w*/*v*) agarose gel, stained with ethidium bromide and photographed under UV light to verify the success of the extraction.

Two regions were amplified directly from the genomic DNA. Fragments of the Translation Elongation Factor 1α (EF) and RNA polymerase second largest subunit (RPB2) were amplified and sequenced using PCR protocols with the following primers: EF1 (5′-ATGGGTAAGGARGACA-3′) and EF2 (5′-GGARGTACCAGTSATCATG -3′) [4]; 5F2 (5′-GGGGWGAYCAGAAGAAGGC-3′), 7cR (5′-CCCATRGCTTGYTTRCCCAT-3′), 7cF (5′-ATGGGYAARCAAGCYATGGG-3′) and 11aR (5′-GCRTGGATCTTRTCRTCSACC-3′) [25,26]. PCR reaction mixtures (total volume 25 μL) contained 2 μL of fungal genomic DNA template, 5 μL PCR buffer (5× green GoTaq reaction buffer), 0.5 μL deoxynucleoside triphosphate (dNTPs), 0.2 μL of GoTaq G2 DNA polymerase and 0.25 μL of each primer. The condition for thermal cycler consists of an initial denaturation step at 94 °C for 2 min, followed by 35 cycles of denaturation at 94 °C for 20 s, annealing at 61 °C for 30 s and extension at 72 °C for 2 min, then a final extension of 72 °C for 7 min.

The fumonisin gene cluster excision site (ΔFGC) was also investigated by PCR as a determinant for the identification of this species. Fvh55 (59-CGCTGCTGTGTGTGGTAACT-39) and Fvh59 (59-AGCTTGTCAACCCAGCAGAT-39) were used as primers [3] and the PCR reaction mixture was prepared as described before. The condition for thermal cycler consists of an initial denaturation step at 94 °C for 2 min, followed by 35 cycles of denaturation at 94 °C for 20 s, annealing at 60 °C for 30 s and extension at 72 °C for 2 min, then a final extension of 72 °C for 7 min. An aliquot of 5 μL of amplified products was separated by electrophoresis onto a 1.5% (*w*/*v*) agarose gel, stained with ethidium bromide and photographed under UV light to observe the result of the amplification. DNA was quantified using 1 kb plus NEB ladder by comparing fluorescence intensity of a known amount of ladder as specified by NEB guidelines. Appropriate amount of PCR product was obtained for further purification and Sanger sequencing with each primer was used during previous amplification. Sanger sequencing service was performed by Eurofins Genomics, Vimodrone (Italy).

### 2.3. Phylogenetic Analysis

A consensus sequence was computed from the forward and reverse sequences using Geneious software (Version: 2020.2). Phylogenetic analyses of our collection were conducted on the sequences of two protein-encoding nuclear genes: translation elongation factor 1α and RNA polymerase second largest subunit (RPB2). Sequence of genes were obtained from published articles covering *Fusarium fujikuroi* species complex diversity [3,27,28,29]. Sequences were aligned using Muscle plugin of Geneious and manually checked and concatenated. A consensus phylogenetic tree of major *Fusarium* species including the *F. verticillioides* and *F. musae* diversity on a total of 733.047 bases was built applying a Bayesian inference using the MrBayes plugin (Version: 2.2.4) in Geneious software (Version: 2020.2). GTR model was selected based on AKAIKE test implemented in Modeltest 3.2. The consensus tree was obtained by 110,000 generations sampling every 200 generation and burn in of 100,000 initial generations.

### 2.4. Antifungal Susceptibility

Nineteen *F. musae* and two *F. verticillioides* strains were tested for in vitro susceptibility to five medical antifungals—itraconazole, voriconazole, posaconazole, isavuconazole and amphotericin B (all Sigma-Aldrich, St. Louis, MO, USA)—and to eight DMIs used for crop protection—prochloraz, tebuconazole, epoxiconazole, difenoconazole, propiconazole, tetraconazole, flusilazole and fenbuconazole (all Sigma-Aldrich). Susceptibility was performed with broth microdilution method according to the Clinical and Laboratory Standards Institute (CLSI) guidelines for filamentous fungi (Reference CLSI M38-A2). All molecules were prepared at final concentrations ranging from 0.03 to 16 mg/L. Broth microdilution assay was performed in RPMI-1640 with glutamine, without bicarbonate (Sigma-Aldrich, St. Louis, MO, USA). Inoculum suspensions were prepared from 2–5-day-old cultures. The conidia suspensions were counted in a haemocytometer chamber and diluted to a final working inoculum of 0.5–5 × 10^4^ CFU/mL. Plates were incubated at 28 °C for 48 h. The minimum inhibitory concentration (MIC) value was the concentration of drug yielding no fungal growth at visual reading: no mycelium was visible, and the medium appeared crystal clear by looking through naked eye. Tests were performed in duplicate. Reference strains *Candida parapsilosis* ATCC 22019 and *Candida krusei* ATCC 6258 were used as quality controls.

### 2.5. Statistical Analysis

Statistical analysis was performed using JAPS software v0.10.2 considering the confidence interval of 95% as significant. Bayesian Mann-Whitney T-test sample was used to assess the discriminatory effect of each fungicide on the origin of the strain.

## 3. Results

### 3.1. Strain Classification

**Figure 1:**
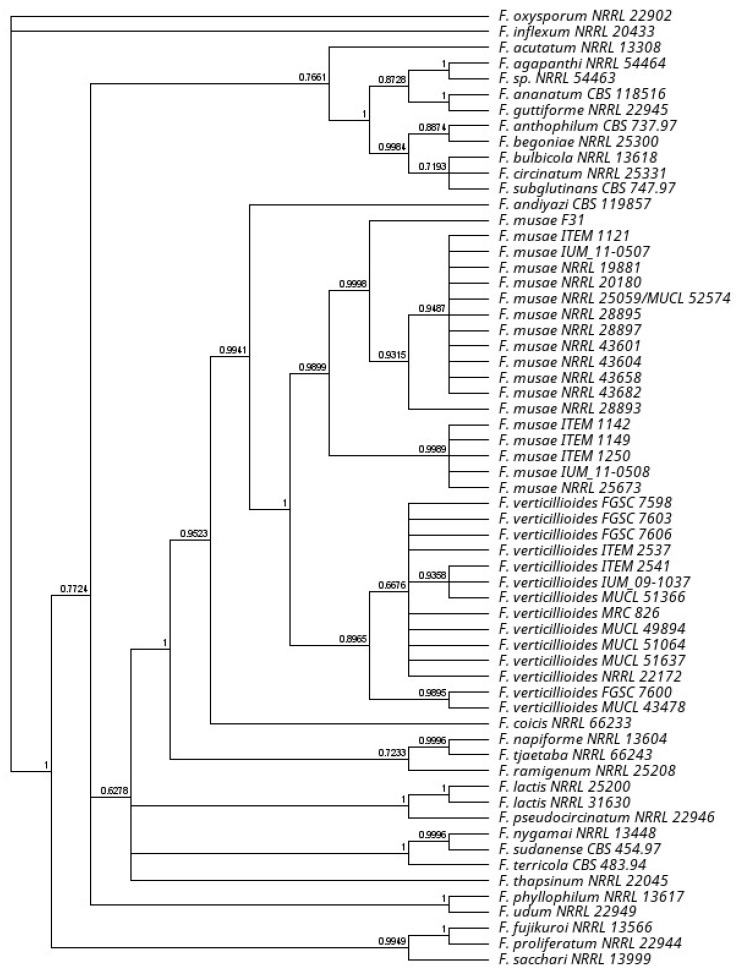
Phylogenetic tree position *F. musae* strains on a *Fusarium* tree based on EF1 and RPB2 sequence diversity. Bayesian posterior probability tree obtained with mr.Bayes plugin in the Geneious software. Branch labels are shown as a measure in support of the nodes.

Four strains isolated in our laboratories from banana fruits and human patients (respectively F31 and IUM 11-0507, IUM 11-0508, IUM 09-1037, Table 1 *) identified at first as *Fusarium musae* were further investigated in this work. Their initial identification was verified using an EF-1 and RPB2 sequencing approach. Their phylogenetic position was observed in relation to other *F. musae* strains and other *Fusarium* species (Figure 1) confirming position of all the *F. musae* strains as a clade close to *F. verticillioides*. The use of fumonisin excision primers confirmed the species attribution of the *F. musae* strains investigated in this work (Supplementary Figure S1) and to confirm reclassification of IUM 09-1037 as *F. verticillioides* that grouped with *F. verticillioides* in the phylogenetic tree.

### 3.2. Antifungal Susceptibility

A total of 21 *Fusarium* isolates, namely 19 *F. musae* (8 of human origin and 11 of vegetable origin), two *F. verticillioides* (one of human origin and one of plant origin) were tested for in vitro antifungal susceptibility. All isolates had good growth capacity at 28 °C, while we observed for *F. musae* a slow growth at 37 °C. For this reason, we chose an incubation at 28 °C for 48 h for all strains tested. The distribution of MIC values and the geometric mean of the MICs (G-MICs) for the antifungals tested are shown in Table 2.

High MICs of itraconazole were observed (G-MIC = 16 mg/L) for both human and plant origin strains of *F. musae*. The MICs of the other three medical azoles showed lower values: the G-MICs ranged from 0.54 mg/L of posaconazole to 1.2 mg/L of voriconazole and to 2.4 mg/L of isavuconazole. No statistically significant differences between the isolates of different origins were observed. The polyene amphotericin B showed a G-MIC of 1.93 mg/L on *F. musae*.

Among the antifungals of agricultural use, the highest activity was shown by the imidazole prochloraz and the triazole tebuconazole (G-MIC of 0.14 mg/L and 0.83 mg/L respectively). The MICs of tebuconazole against the plant origin strains ranged between 0.5 and 1 mg/L (G-MICs = 0.73), slightly lower than those of clinical strains (range 1–2 mg/L). An identical G-MIC (3.58 mg/L) was observed for difenoconazole and propiconazole with a slightly wider range for difenoconazole (2–8 mg/L) than for propiconazole (2–4 mg/L). Tetraconazole and fenbuconazole showed G-MICs > 16 mg/L for all the 19 *F. musae* strains.

The G-MIC of flusilazole for the 19 *F. musae* was 1.55 mg/L, ranging from 1 to 2 mg/L for both human and plant origin isolates. The same range of MIC values was observed for epoxiconazole (G-MIC = 1.11 mg/L), with a MIC value of 1 mg/L for all the 11 isolates of plant origin.

The antifungal susceptibility profile of *F. musae* isolates was different from that of *F. verticillioides* isolates with higher MIC values of epoxiconazole, propiconazole, flusilazole and fenbuconazole (1.11 vs. 0.31, 3.58 vs. 0.31, 1.55 vs. 0.37, >16 vs. 1 mg/L, respectively).

Different MICs of itraconazole were observed between the clinical strain (MIC = 16 mg/L) and the plant strain (MIC = 0.5 mg/L) of *F. verticillioides*. On the contrary, similar MICs of the other medical triazoles were observed between the strains of different origin for isavuconazole, voriconazole and posaconazole. Prochloraz is confirmed to be the agricultural fungicide with lower MIC values, also for *F. verticillioides*.

Origin of the isolate (human or banana) showed a significant difference only for difenoconazole (BF10 3.988) and tebuconazole (BF10 1.911) susceptibility. In both cases strains from banana had higher susceptibility compared to the human strains (Supplementary Table S1).

## 4. Discussion

Fungicide susceptibility varies widely in the genus *Fusarium*. Our work is a step towards understanding the patterns of variability within the genus focusing on an emerging inter-kingdom species such as *F. musae*. These results can be used to infer causes of fungicide resistance relevant to inter-kingdom epidemiology, as well as to provide information to medical practitioners as to which fungicides or medicines are likely to be effective in a given clinical setting. To obtain epidemiological information useful for the clinical practice, fungicide sensitivity studies are coupled to accurate identification of the strains.

In Europe, *F. fujikuroi* species complex, to which *F. verticillioides* and *F. musae* belong, is the predominant cause of human deep infections, and in Italy *F. verticillioides* is the most prevalent species [11,30]. This makes its investigation even more significant. The re-identification of one of the strains used in the study, by sequencing the RPB2 gene in addition to the EF, highlights the importance of performing a multilocus molecular identification especially for those species that are morphologically hard to distinguish, such as *F. verticillioides* and *F. musae* [31].

Azoles are a widely used category of fungicides employed against human fusariosis as well as plant fungal infections. In this study we focused on the antifungal activity of 13 azoles, chosen among the most used azoles in both clinical and agricultural field, against *F. musae* and *F. verticillioides* isolated from human and plant samples. The results of our work are comparable with data already present in literature [18,32,33,34]. Only MIC values obtained by [10] seem to be higher, probably because of a different incubation timing. As already demonstrated the spectrum and the power of each azole derivative is unique and different from the others, this implies that different drugs have different safety profiles and also antifungal activity as we could observe from the MIC values we obtained. In particular, we observed that voriconazole, isavuconazole and mostly posaconazole may be effective against these two *Fusarium* species studied. On the contrary, itraconazole showed no effect against *F. musae* and *F. verticillioides*, confirming previous studies [10,30,32]. These data may contribute to improving the decision on the selection of the most appropriate molecule to be used in a clinical setting where a given antifungal might encounter a resistant infecting strain. Our results contribute to improving the knowledge on the risk of potential resistance occurrence within the Fusarium genus.

Little is known about the activity of azoles used in crops, especially against *Fusarium fujikuroi* species complex. According to the European Union for crop protection [35] the imidazole prochloraz is one of the most used in the fields and it appears to be the most effective also in our in vitro study against both *F. verticillioides* and *F. musae*. Tetraconazole and fenbuconazole, instead, showed no in vitro activity against the two species. A poor activity of tetraconazole and fenbuconazole had also been observed in experiments conducted on seeds damaged by fusariosis, on which treatment with the two drugs proved ineffective [36]. Among the triazoles for agricultural use, on the other hand, tebuconazole has definitely shown the best in vitro activity against both *F. musae* and *F. verticillioides*.

If we compare sensitivity of the two sister species *F. verticillioides* and *F. musae*, we identified some differences in susceptibility to the azoles. In particular, epoxiconazole, propiconazole, flusilazole and fenbuconazole seemed to need higher concentrations to inhibit the growth of *F. musae* than those needed for its sister species, causing suspicion of some kind of resistance—intrinsic or induced by exposure to these antifungals. Given the small number of strains of *F. verticillioides* these results should suggest future work with a large and diverse population of *F. verticillioides*. Nonetheless our data are largely consistent with previous literature dealing with azole sensitivity in *F. verticillioides*, showing low *F. verticillioides* MIC values have been observed for posaconazole (range 0.25–0.5 mg/L) and voriconazole (0.5–2 mg/L) [11,37]. One exception is the data obtained by Triest [10] on *F. verticillioides* showing higher MIC for some azoles. These differences could be partly attributable to Triest’s use of the EUCAST method. On the other hand, previous studies [11] conducted with the EUCAST method were consistent with our observation.

Since azoles are the main treatment for both human and agricultural fungal diseases, a major concern could be the predictable emergence of cross-resistance to clinical isolates, driven by the massive use of azole fungicides in agriculture, which have the same mechanism of action as that of those used in humans, as already known for *Aspergillus fumigatus* [38,39]. As in *A. fumigatus* and in other fungi, mutations or overexpression of CYP51 gene, encoding 14α-demethylase, is involved in azole resistance, similar mechanisms might be responsible for induced azole resistance also in *Fusarium*.

Pujol I. et al. [40], who compared different *Fusarium* spp. strains of clinical and environmental origin against clinical antifungal drugs, observed a similar activity against both groups of fungi. Moreover, in the present study, analysing the origin of the isolate, human or vegetable (banana), mostly no significant differences were found. The only difference was observed for two agricultural triazoles, difenoconazole and tebuconazole, for which the human *F. musae* isolates showed a reduced susceptibility. In most fungi, clinical isolates are thought to be more resistant to antifungals than environmental isolates, probably due to antifungal exposure during therapy in chronically ill patients [18]. Further studies on larger population are needed to verify the consistency of this finding. Understanding the evolutionary pressure of environmental fungicides also on clinical isolates may allow to better decipher the mechanisms leading to fungicide adaptation [41]. This can lead to set the most appropriate clinical and agricultural fungicide treatments under the “One health” framework applied to *Fusarium* pathogen control strategies [2].

## 5. Conclusions

Our work defines for the first time a susceptibility level of *F. musae* strains obtained from different hosts and different continents for 12 azoles and amphothericin B, providing the basis for monitoring evolution of the pathogen sensitivity to fungicides both in clinical and agricultural settings.

## Figures and Tables

**Table 1:** Collection of strains of *F. musae* and *F. verticillioides* used in this work. The name of each strain, its species, country of origin, host species and tissue type, [references] and GenBank accession numbers for TEF-1α and RPB2 are reported.

Strain	Species	Country	Host (Tissue)	Reference	NCBI Accession Number TEF-1α	NCBI Accession Number RPB2
F31	*F. musae*	Dominican Republic	Banana (fruit)	[23]	MW916961 ^a^	MW916958 ^a^
IUM 11-0507	*F. musae*	Greece	Human (blood)	[8]	MW916959 ^a^	MW916956 ^a^
IUM 11-0508	*F. musae*	Greece	Human (cornea)	[8]	MW916960 ^a^	MW916957 ^a^
NRRL 28893	*F. musae*	Mexico	Banana (fruit)	[3]	FN552092	FN552114
NRRL 28895	*F. musae*	Mexico	Banana	[3]	AF273314.1	MZ346032 ^a^
NRRL 28897	*F. musae*	Mexico	Banana	[14]	AF273316.1	MZ346033 ^a^
NRRL 43601	*F. musae*	Maryland, USA	Human (skin)	[15]	MZ346030 ^a^	EF470191
NRRL 43604	*F. musae*	Ohio, USA	Human (nasal sinus)	[15]	MZ346031 ^a^	EF470194
NRRL 43658	*F. musae*	Minnesota, USA	Human (contact lens)	[15]	EF452989	EF470028
NRRL 43682	*F. musae*	Minnesota, USA	Human (cornea)	[15]	EF453009	EF470048
NRRL 25673	*F. musae*	Guatemala	Banana (fruit)	[3]	FN552091	FN552113
NRRL 25059 (MUCL 52574) *	*F. musae*	Honduras	Banana (fruit)	[3]	FN552086	FN552108
IHEM 20180	*F. musae*	Brussels, Belgium	Human (sinus biopsy)	[10]	KJ865533	KM582792
IHEM 19881	*F. musae*	Brest, France	Human (shoulder biopsy)	[10]	KJ865532	KM582791
ITEM 1121	*F. musae*	Panama	Banana (fruit)	[3]	FN552093	FN552115
ITEM 1142	*F. musae*	Ecuador	Banana (fruit)	[3]	FN552094	FN552116
ITEM 1149	*F. musae*	Panama	Banana (fruit)	[3]	FN552095	FN552117
ITEM 1250	*F. musae*	Canary Islands	Banana (fruit)	[3]	FN552090	FN552112
IUM 09-1037	*F. verticillioides*	Italy	Human (blood)	[8]	MW915565 ^a^	MW915564 ^a^
MUCL 43478	*F. verticillioides*	Kansas, USA	Corn	[3]	FN552074	FN552096

* The strain was obtained from two culture collections. ᵃ Sequenced and deposited as part of this work.

**Table 2:** In vitro susceptibility to antifungal drugs of the 21 *Fusarium* isolates determined by broth microdilution according to the Clinical and Laboratory Standards Institute (CLSI) methodology.

*Fusarium* (No. of Tested Isolates)	Antifungal	Isolates Origin	No. of Isolates with MIC (mg/L) of:											^a^ G-MIC	^b^ MIC_50_	^c^ MIC_90_
			0.03	0.06	0.12	0.25	0.5	1	2	4	8	16	>16			
*F. musae* (19)	Isavuconazole	total						2	10	7				2.4	2	4
		C (8)						2	4	2				2	2	4
		V (11)							6	5				2.74	2	4
	Itraconazole	total										5	14	16	>16	>16
		C (8)											8	16	>16	>16
		V (11)										5	6	16	>16	>16
	Posaconazole	total				3	11	5						0.54	0.5	1
		C (8)					4	4						0.71	0.5	1
		V (11)				3	7	1						0.44	0.5	1
	Voriconazole	total						14	5					1.2	1	2
		C (8)						6	2					1.19	1	2
		V (11)						8	3					1.21	1	2
	Amphotericin B	total						1	18					1.93	2	2
		C (8)						1	7					1.83	2	2
		V (11)							11					2	2	2
	Difenoconazole	total							5	12	2			3.58	4	4
		C (8)							2	4	2			4	4	8
		V (11)							3	8				3.31	4	4
	Epoxiconazole	total						16	3					1.11	1	2
		C (8)						5	3					1.29	1	2
		V (11)						11						1	1	1
	Fenbuconazole	total										3	16	>16	>16	>16
		C (8)											8	>16	>16	>16
		V (11)										3	8	>16	>16	>16
	Flusilazole	total						7	12					1.55	2	2
		C (8)						1	7					1.83	2	2
		V (11)						6	5					1.37	1	2
	Propiconazole	total							3	16				3.58	4	4
		C (8)							1	7				3.67	4	4
		V (11)							2	9				3.53	4	4
	Tebuconazole	total					5	13	1					0.83	1	1
		C (8)						7	1					1.09	1	1
		V (11)					5	6						0.73	1	1
	Tetraconazole	total											19	>16	>16	>16
		C (8)											8	>16	>16	>16
		V (11)											11	>16	>16	>16
	Prochloraz	total			15	4								0.14	0.12	0.25
		C (8)			5	3								0.16	0.12	0.25
		V (11)			10	1								0.13	0.12	0.12
*F. verticillioides* (2)	Isavuconazole	total						1	1					1.5	1	2
		C (1)							1							
		V (1)						1								
	Itraconazole	total					1						1	8.25	0.5	>16
		C (1)											1			
		V (1)					1									
	Posaconazole	total					2							0.5	0.5	0.5
		C (1)					1									
		V (1)					1									
	Voriconazole	total						2						1	1	1
		C (1)						1								
		V (1)						1								
	Amphotericin B	total						1	1					1.5	1	2
		C (1)						1								
		V (1)							1							
	Difenoconazole	total							2					2	2	2
		C (1)							1							
		V (1)							1							
	Epoxiconazole	total			1		1							0.31	0.12	0.5
		C (1)					1									
		V (1)			1											
	Fenbuconazole	total					1		1					1	0.5	2
		C (1)							1							
		V (1)					1									
	Flusilazole	total				1	1							0.37	0.25	0.5
		C (1)					1									
		V (1)				1										
	Propiconazole	total				1	1							0.37	0.25	0.5
		C (1)					1									
		V (1)				1										
	Tebuconazole	total					1	1						0.75	0.5	1
		C (1)						1								
		V (1)					1									
	Tetraconazole	total						1					1	8.5	1	>16
		C (1)											1			
		V (1)						1								
	Prochloraz	total			1	1								0.18	0.12	0.25
		C (1)			1											
		V (1)				1										

^a^ G-MIC, geometric mean MIC. ^b^ MIC50: MIC at which 50% of isolates are inhibited. ^c^ MIC90: MIC at which 90% of isolates are inhibited. C = clinical origin. V = vegetable origin.

## Data Availability

Sequences are available in NCBI public database. Raw data are available upon request to the corresponding author.

## Supplementary Materials

The following are available online at https://www.mdpi.com/article/10.3390/jof7090784/s1, Figure S1. PCR amplification with primers Fvh55 and Fvh59, Table S1: Bayesian independent Mann-Whitney t-test calculated on the differential response of strains originating from banana fruits and human patients.
