# Supplementary material for: Fusarium musae from Diseased Bananas and Human Patients: Susceptibility to Fungicides Used in Clinical and Agricultural Settings
Source: J Fungi (Basel). 2021 Sep 21;7(9):784. doi: 10.3390/jof7090784 (PMC8467134; doi:10.3390/jof7090784)

Supplementary

Figure S1. Figure 1. PCR amplification with primers Fvh55 and Fvh59. Successful amplification of the fumonisin gene cluster excision site (ΔFGC) represents the inability of the strain to produce the toxin fumonisin, a characteristic trait of *F. musae* species. If amplification occurs (1178 bp) the cluster is not present. Loading order is: (1) IUM11-0507, (2) IUM11-0508, (3) IUM09-1037 and (4) F31; (5) *Ciborinia camelliae* ITAC2 (negative control), (6) water. Marker 1kb on the left.

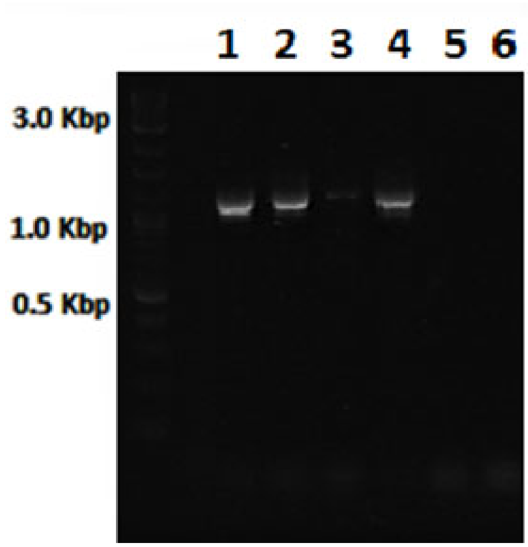

Supplement: Supplementary file 1 [file jof-07-00784-s001.zip › jof-1383643-supplementary/Supplementary figure.pdf]
