# Supplementary material for: Fusarium musae from Diseased Bananas and Human Patients: Susceptibility to Fungicides Used in Clinical and Agricultural Settings
Source: J Fungi (Basel). 2021 Sep 21;7(9):784. doi: 10.3390/jof7090784 (PMC8467134; doi:10.3390/jof7090784)
Supplement: Supplementary file 1 [file jof-07-00784-s001.zip › jof-1383643-supplementary/Table S1_new.pdf]

Table S1

Bayesian independent Mann-Whitney t-test calculated on the differential response of strains originating from banana fruits and human patients.

	BF_{10}	W	R^
ITRA	0.530	51.00	1.000
VORI	0.475	52.00	1.000
POSA	0.472	36.00	1.000
ISAV	0.542	52.50	1.000
AMB	0.474	39.00	1.001
EPOXICONAZ	0.587	56.00	1.000
DIFENOCONAZ	3.988	78.50	1.000
PROPICONAZ	1.911	69.50	1.001
TETRACONAZ	1.076	64.00	1.001
FLUSILAZ	0.540	51.50	1.001
FENBUCONAZ	1.291	69.50	1.000
* PROCHLORAZ/ TEBUCONAZ	-	-	-

Note. Result based on data augmentation algorithm with 5 chains of 1000 iterations.

*The variance in PROCHLORAZ and TEBUCONAZ is equal to 0 after grouping based on origin of the sample
